# Antiproliferative and cell cycle arrest potentials of 3-O-acetyl-11-keto-β-boswellic acid against MCF-7 cells in vitro

**DOI:** 10.1186/s43141-023-00529-2

**Published:** 2023-07-02

**Authors:** Saja A. Ahmed, Ahmed F. Al-Shanon, Ali Z. Al-Saffar, Alene Tawang, Jameel R. Al-Obaidi

**Affiliations:** 1 Department of Molecular and Medical Biotechnology, College of Biotechnology, Al-Nahrain University, Baghdad, Iraq; 2 Biotechnology Research Center, Al-Nahrain University, Baghdad, Iraq; 3 Department of Biology, Faculty of Science and Mathematics, Universiti Pendidikan Sultan Idris, 35900 Tanjong Malim, Perak Malaysia

**Keywords:** Acetyl-11-keto-β-boswellic acid, Apoptosis, Cytotoxicity, MCF-7

## Abstract

**Introduction:**

Cancer is a major issue in medical science with increasing death cases every year worldwide. Therefore, searching for alternatives and nonorthodox methods of treatments with high efficiency, selectivity and less toxicity is the main goal in fighting cancer. Acetyl-11-keto-β-boswellic acid (AKBA), is a derivative pentacyclic triterpenoid that exhibited various biological activities with potential anti-tumoral agents. In this research, AKBA was utilized to examine the potential cytotoxic activity against MCF-7 cells in vitro and monitor the cellular and morphological changes with a prospective impact on apoptosis induction.

**Methods:**

The cytotoxic activity of AKBA was measured by 3(4,5dimethylthiazole- 2-yl)-2,5 diphyneltetrazolium bromide (MTT) assay. A dose-dependent inhibition in MCF-7 cell viability was detected. The clonogenicity of MCF-7 cells was significantly suppressed by AKBA increment in comparison with untreated cells.

**Result:**

Morphologically, exposure of MCF-7 cells to high AKBA concentrations caused changes in cell nuclear morphology which was indicated by increasing in nuclear size and cell permeability intensity. The mitochondrial membrane potential (ΔΨm) was reduced by increasing AKBA concentration with a significant release of cytochrome c. Acridine orange/ethidium bromide dual staining experiment confirmed that MCF-7 cells treated with AKBA (IC50 concentration) displayed a late stage of apoptosis indicated by intense and bright reddish colour.

**Conclusion:**

A significant increase in reactive oxygen species formation was observed. Caspase 8 and caspase 9 activities were estimated and AKBA activated the production of caspase 8 and caspase 9 in a dose-dependent pattern. Finally, the cell phase distribution analysis was conducted, and flow cytometric analysis showed that AKBA at 200 μg mL-1 significantly arrest MCF-7 cells at the G1 phase and triggered apoptosis.

## Introduction

Cancer is abnormal cell growth that proliferates in an uncontrolled pattern that allows it to continue and spread. Changes in cell cycle control genes, oncogene activation and anti-oncogene mutations lead to the expression involved in cell-division stimulating proteins [15]. Since cancer is still a major serious health issue in the world, many studies focusing on alternative medicine and complementary remedies are being pursued. Breast cancer is considered the highest common type of cancer among women around the globe and is associated with dominant morbidity and mortality in women [4]. In Iraq, breast cancer considers the most prevalent malignant neoplasm with a devastating death rate (24%) among Iraqi women and the second most deadly type of cancer (12%) among the Iraqi population [9]. Phytochemicals extracted from medicinal herbs have been used as active medications for the avoidance and therapy of different health complications [5, 43]. Plant natural products were recorded for their effectiveness to treat various health issues and chronic diseases [42]. Within the last few decades, medicinal plants became in consideration for the treatment of cancer because of their anti-neoplastic activity and to some extent safety [28]. Previous reports showed that several plants contain rather highly bioactive phytochemicals with promising uses as anticancer drugs [47, 51].

Pentacyclic triterpenoids are a bioactive compound found in the plant kingdom. They have great importance as a source of potential therapy for various health complications as well as potential anti-cancer [44]. Many biological activities of pentacyclic triterpenoids were studied, for instance, anti-inflammatory, apoptosis and cell cycle arrest, their effect on angiogenesis and others [16].

The extract of the gum resin of the plant *Boswellia* has been utilized widely in complementary medicine and in treating various diseases for instance arthritis, ulcers, asthma and inflammatory illnesses [54]. *Boswellia* extracts contain a mix of the essential lipophilic part with different ratios depending on the species (e.g. *serrata, sacra*, *ameero*) [18]. Triterpenoids within the lipophilic part are known as boswellic acids which are the main entities of the resin [20].

Boswellic acid is a combination of pentacyclic triterpenoid compounds which include the following: 11-k3-O-acetyl-11-keto-β-boswellic acid (AKBA) and eto-β-boswellic acid (KBA). Accordingly, drug discovery research focused mainly on these two forms [41]. Boswellic acids are responsible for most of the therapeutic properties of *Boswellia*. The medicinal potentials of boswellic acids such as antioxidant, anti-inflammatory, immune system modulation, antihyperlipidemic and antiobesity attributes have been described [11]. Previous and recent studies have revealed compelling inhibitory activities of boswellic acids toward different types of tumour cells like colon, lung, prostate, leukaemia and others [2, 35, 36]. Nevertheless, the exact mechanism of exerting the antiproliferative effect and apoptosis induction by boswellic acids against tumour cells is still under research.

The numerous biological activities of AKBA and the possibility to pursue being an alternative and/or complementary therapy against different types of cancer require a series of AKBA toxicity examinations before release and utilizing a new drug. In this study, the antiproliferative effect and apoptosis-inducing capabilities of AKBA against breast tumour MCF-7 cells were evaluated in vitro. Morphological changes in MCF-7 cells, caspase activation and cell death mechanism were also investigated in this study.

## Materials and methods

### Materials

AKBA (>98.0% purity), doxorubicin, acridine orange, ethidium bromide and all materials used in tissue culture experiments were obtained from Sigma-Aldrich (St. Louis, USA). The 3(4,5-dimethylthiazole-2-yl)-2,5 diphenyltetrazolium bromide (MTT) kit was obtained from Intron Biotech (Korea). DNA reagent CycleTEST™ PLUS kit was obtained from BD Biosciences (USA), while Caspase-Glo® 9 and 8 were obtained from Promega (USA). Multiparametric Cellomics cytotoxicity 3 kit and Cellomics oxidative stress kit were obtained from Thermo Fisher Scientific (USA). Dimethyl sulfoxide was used to dissolve AKBA at a concentration of 10 mg mL^−1^ and stored at 4 °C until used. DMSO was used as a negative control with a final concentration of 0.1% in culture experiments. Reagents used in this study were of analytical grade, and solutions were prepared using highly purified water.

### Cell line and culture maintenance

Breast adenocarcinoma MCF-7 and normal epithelial breast MCF-10A cell lines were kindly supplied by Biotechnology Research Center et al.-Nahrain University. Cells were kept in RPMI medium supplemented with 10% foetal bovine serum (heat-inactivated), 2-mM L-glutamine, 20-mM HEPES, 100 U mL^−1^ penicillin G and 100 µg mL^−1^ streptomycin. Cells were seeded in tissue culture flasks and allowed to reach about 90% monolayer confluency after 24–48 h incubation at 37 °C augmented with 5% CO_2_. For subculturing, gentle trypsinization uses trypsin (2–3 mL, 50 mg mL^−1^) to harvest the cells [37].

### MTT assay (in vitro cytotoxicity)

The cytotoxic activity of AKBA was assessed using an MTT assay against MCF-7 and MCF-10A cells. Cells were seeded into a flat 96-well plate (100 µL per well at a concentration of 1 × 10^6^ cells per well) and allowed to proliferate for 24 h at 37 °C in CO_2_ (5%) incubator. Following incubation, the medium was replaced by a fresh medium containing increasing treatments of AKBA (12.5, 25, 50, 100, 200 and 400 µg mL^−1^) and was added for each well. The plate was further incubated for another 24 h. Following incubation, 10 µL of MTT solution was added to each well. The plate was incubated for another 4 h. The medium and MTT were discarded, and 100 µL of solubilization solution (DMSO) was added to solubilize the formazan crystals. When the solubilization process of purple formazan complete, optical density at 570 nm was measured using a plate reader (Bio-Rad, USA), and inhibition percentage was calculated concerning vehicle control (untreated cells). Treatments were done in triplicate, and a half-maximal inhibitory concentration (*IC*_50_) value was calculated for each cell line [22].

### Clonogenic assay

MCF-7 clone formation’s inhibitory level was investigated as described previously [50]. Cells were seeded into 6-well plates with a concentration of 2 × 10^5^ cells mL^−1^. Then cells were treated with AKBA (50, 100 and 200 µg mL^−1^) and incubated for 48 h at 37 °C and atmospheric air supplied with 5% CO_2_. Cells were gently trypsinized and seeded at a concentration of 10^3^ cells per well of a 6-well plate and left to grow for about 7 days until DMSO-treated cells reached 90% confluency. Cells were washed twice with PBS and stained with crystal violate solution (1% crystal violate in 25% methanol) for 1 h at 26 °C. Plates were washed with distilled water and dried before analysis using ImageJ software. Data are representative of the standard deviation (SD) of three independent experiments.

### Cellomics multiparameter cytotoxicity

Morphological and cellular changes in MCF-7 cells were detected in high-content screening (HCS) (ArrayScan, Thermo Scientific, USA) using Cellomics multiparameter cytotoxicity 3 kits as described earlier [3]. The kit measures independent parameters related to cell morphological changes including viable cell count, mitochondrial membrane potentials (ΔΨm), cell permeability, nuclear intensity and cytochrome *c*. MCF-7 cells (1 × 10^5^ per well) were seeded into a flat 48-well plate and incubated for 24 h at 37 °C, 5% CO_2_. Cells were treated with AKBA (50, 100 and 200 µg mL^−1^), doxorubicin treatment (1 mM, positive vehicle) and untreated cells (negative control) and incubated for another 24 h. Next, ΔΨm dye (excitation 552/emission 576) and cell permeability dye (excitation 491 nm/emission 509 nm) were applied on MCF-7 cells and allowed to incubate for 1 h at 37 °C. Later, cells were subjected to fixation, permeabilization and blocking with bovine serum albumin (3%) before probing with cytochrome *c* primary mouse antibody for 1 h. After three washing steps with PBS, goat anti-mouse secondary IgG conjugated with DyLight™ 649 was added and incubated for 1 h. Cells were rinsed with PBS containing 1% Tween-20, and then Hoechst 33,258 (excitation 330 nm/emission 420 nm) was utilized to stain the nuclei. Finally, the Cellomics ArrayScan HCS Reader (Thermo Scientific, USA) was used to identify and visualize fluorescently stained cells and detect the fluorescent intensity. In each well, 1000 cells were analysed, and fluorescent images were acquired using suitable filters. The average intensity for each parameter was obtained as mean ± SD of three independent sets of experiments.

### Deal staining of acridine orange-ethidium bromide (AO/EtBr)

The AO/EtBr dual staining procedure was used to monitor the death of tumour cells and apoptosis induction. MCF-7 cells were seeded at a concentration of 1 × 10^5^ mL^−1^ per well into a 96-well microtiter plate and incubated overnight at 37 °C, 5% CO_2_. After incubation, cells were treated with AKBA (*IC*_50_ concentration, ~ 100 µg mL^−1^) and allowed the cells to incubate for another 24 h. After that, the cells were rinsed twice with PBS to remove the medium and an aliquot of 100 µL of dual fluorescent dye (equal volume of AO and EtBr, 10 µg mL^−1^) was added to each well and then visualizing the cells under a fluorescence inverted microscope. Identification criteria were concentrated green areas of chromatin condensation in the nucleus considered early apoptosis, green intact nuclei considered viable cells and dense orange areas of chromatin condensation and intact nuclei considered late apoptosis [7].

### ROS production assay

Reactive oxygen species generation was detected in MCF-7 cells treated with AKBA (50, 100 and 200 µg mL^−1^) using Cellomics oxidative stress 1 HCS Reagent Kit. MCF-7 (1 × 10^4^ per well) were seeded into 96-well plates for 24 h under conditions of 37 °C and 5% CO_2_. The medium was replaced with fresh medium containing AKBA treatments were applied and compared with doxorubicin (1 mM)-treated cells (positive vehicle) and DMSO-treated cells (negative control). After 24 h, 50 µL of staining solution (500-nM Hoechst 33,342 and 2.5 µg mL^−1^ dihydroethidium) was added and incubated for 30 min at 37 °C. Cells were fixed with paraformaldehyde (3.5% in PBS) for 15 min and then rinsed with PBS. Analysis of stained cells was analysed using a Cellomics ArrayScan HCS Reader (Thermo Scientific, USA). Average fluorescent intensity was measured (excitation 520 nm/emission 620 nm), and data are representative of ± SD of three independent experiments.

### Caspase 8 and 9 activity

The activities of Caspase 8 and 9 were examined using bioluminescent Caspase-Glo® 8 and 9 kits (Promega, USA) following the manufacturer’s instructions. In brief, MCF-7 cells treated with AKBA (50, 100 and 200 µg mL^−1^) for 24 h were subjected to 100 µL of Caspase-Glo® reagent for half an hour at room temperature. The luminescence of each sample was calculated using an ELISA reader (Bio-Rad, USA) at 405 nm. The values are represented mean ± SD and performed in triplicates, and treated samples were compared with positive and negative vehicles.

### Cell cycle analysis

The distribution of MCF-7 cells cycle was analysed using CycleTEST™ kit (BD Biosciences, USA) as previously described [44]. MCF-7 cells (1–5 × 10^5^) were seeded into 12-well plate for 24 h at 37 °C, 5% CO_2_. Following incubation, the medium was aspirated, and cells were treated with AKBA (50, 100 and 200 µg mL^−1^), compared with positive vehicle (1-mM doxorubicin) and negative control (untreated cells). After 24 h, a suspension of MCF-7 cells (1 × 10^6^ cell mL^−1^) was prepared following successive steps of media aspiration, washing, gentle trypsinization and centrifugation. MCF-7 cells were washed with propidium iodide (PI) stain solution and kept in dark for 10 min. Minimally, 1000 cells were analysed for each treatment using FACSCanto II™ flow cytometer (BD Biosciences, USA) emitting at 488 nm (PI excitation). Cell cycle distribution was represented as histograms using ModFit LT (Verity Software House Inc., Topsham, ME, USA) software for three independent sets of experiments.

### Statistical analysis

Given data are mean ± SD, and all experiments were executed in triplicates. Statistical differences between various tested groups were analysed using a one-way analysis of variance ANOVA. Statistical significance was defined as *p ≤ 0.05 or **p ≤ 0.01. Data analysis was performed using GraphPad Prism version 9 (GraphPad Software Inc., La Jolla, CA, USA).

## Results

### Cytotoxic effect of AKBA on MCF-7 cells in vitro

**Fig. 1:**
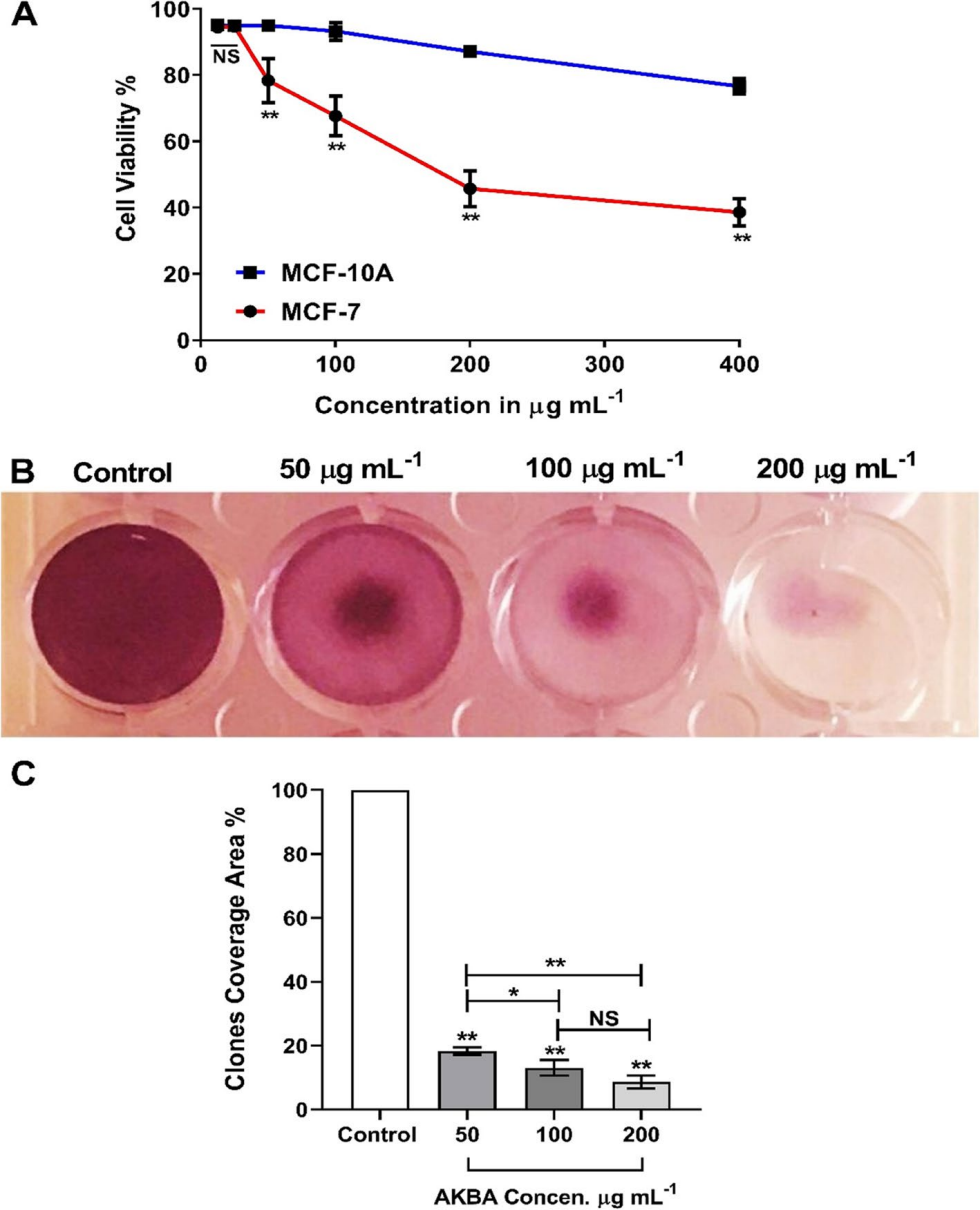
**A** Cell survival curve (mean ± SD%) of MCF-7 cells and MCF-10A after treatment with AKBA using MTT in vitro assay at 37 °C, 5% CO_2_ for 24 h. **B** Clonogenic survival assay for MCF-7 treated with 50, 100 and 200 µg mL.^−1^ AKBA compared with untreated cells (control) at 37 °C, 5% CO_2_ for 7 days (experimental plates). **C** Histogram of mean % (± SD) clones coverage area. *p ≤ 0.05, **p ≤ 0.01, NS nonsignificant, SD standard deviation, *n* = 3

Due to the importance of adopting different compounds isolated from natural sources as complementary medicines for treating many health issues including cancer, the antitumoral effect of AKBA was evaluated against MCF-7 and MCF-10A cells using an MTT assay for 24 h. According to Fig. 1A, AKBA exhibited significant (*p* ≤ 0.0001) antitumoral activity against MCF-7 cells in a dose-dependent pattern with a reduction rate of 21.65 ± 6.63, 32.37 ± 6.97, 54.29 ± 5.35 and 61.42 ± 4.14% for the concentrations 50, 100, 200 and 400 µg mL^−1^, respectively. By comparing with MCF-10A, only AKBA treatments at concentrations 12.5 and 25 µg mL^−1^ showed no significant differences in MCF-7 cell inhibition rate with calculated *IC*_50_ of 101.1 and 275.2 for MCF-7 and MCF-10A, respectively. The antiproliferative effect of AKBA against MCF-7 cells was determined using a clonogenic assay to confirm results obtained from the MTT experiment. The clonogenicity of MCF-7 cells was significantly (*p* ≤ 0.0001) reduced by increasing the concentration of AKBA compared with untreated cells. All treatments revealed a major impact on clones’ formation with a high reduction in MCF-7 cell proliferation with remaining clones’ coverage area of 18.27 ± 1.14, 13.01 ± 2.44 and 8.62 ± 2.00% for treatments 50, 100 and 200 µg mL^−1^, respectively (Fig. 1B and C).

### Multiparametric cytotoxicity of AKBA

**Fig. 2:**
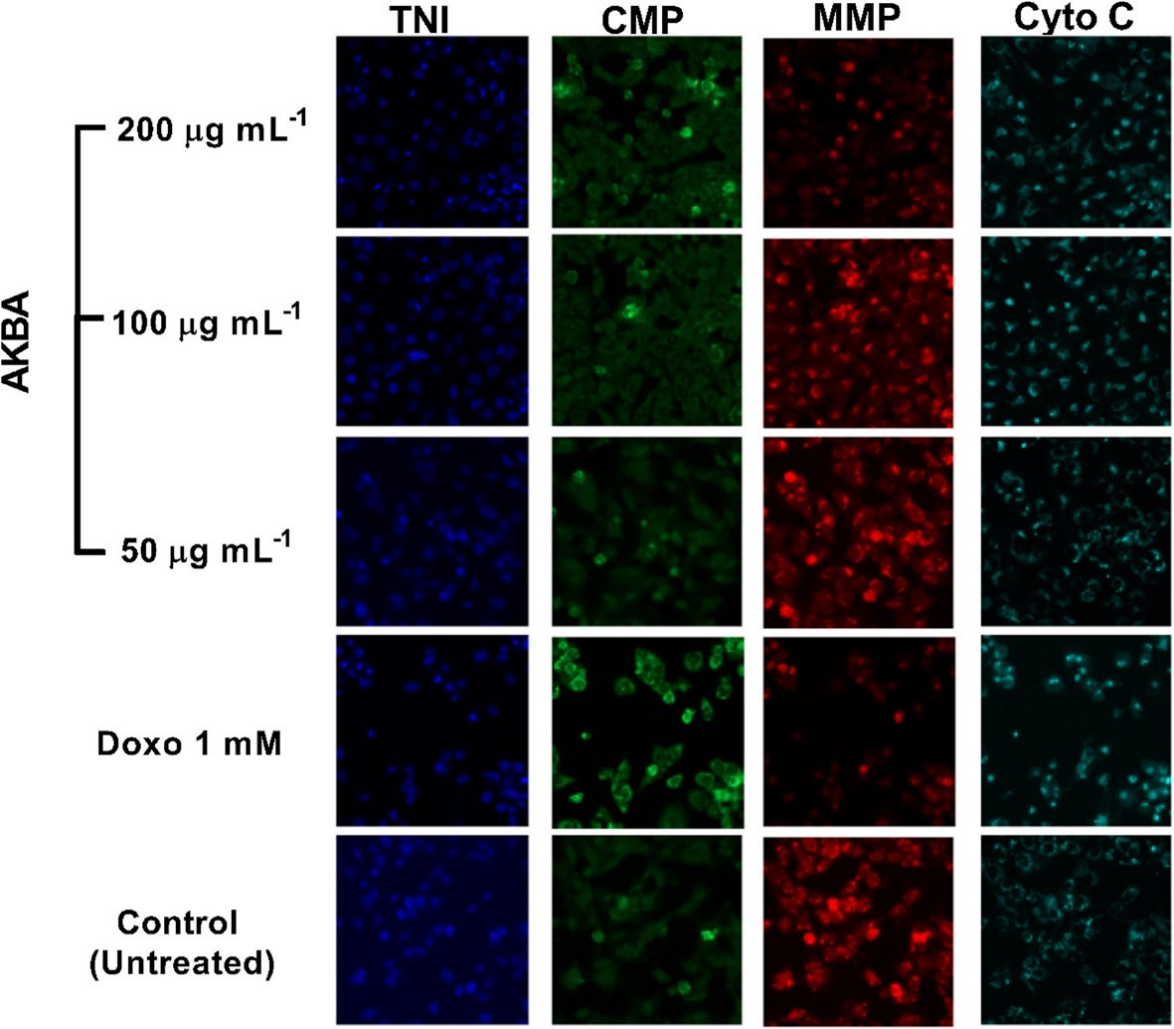
Multiparameter cytotoxic analysis of MCF-7 cells treated with AKBA (50, 100 and 200 µg mL^−1^) after 24 h of incubation at 37 °C. Cells were stained with Hoechst 33,342 (blue) for nuclei, permeability dye (green) for membrane permeability monitoring, ΔΨm dye (red) for mitochondrial membrane potential changes and with goat anti-mouse secondary antibody conjugated with DyLight.™ for cytochrome *c* releasing (magnification: 200 ×)

The cytotoxicity of AKBA was assessed by HCS to detect changes in MCF-7 cells using the nuclear intensity, cell viability, membrane permeability, ΔΨm and cytochrome *c* release as measurement parameters. Images of MCF-7 cells treated with the AKBA, and doxorubicin as well as control cells, are illustrated in Fig. 2.

**Fig. 3:**
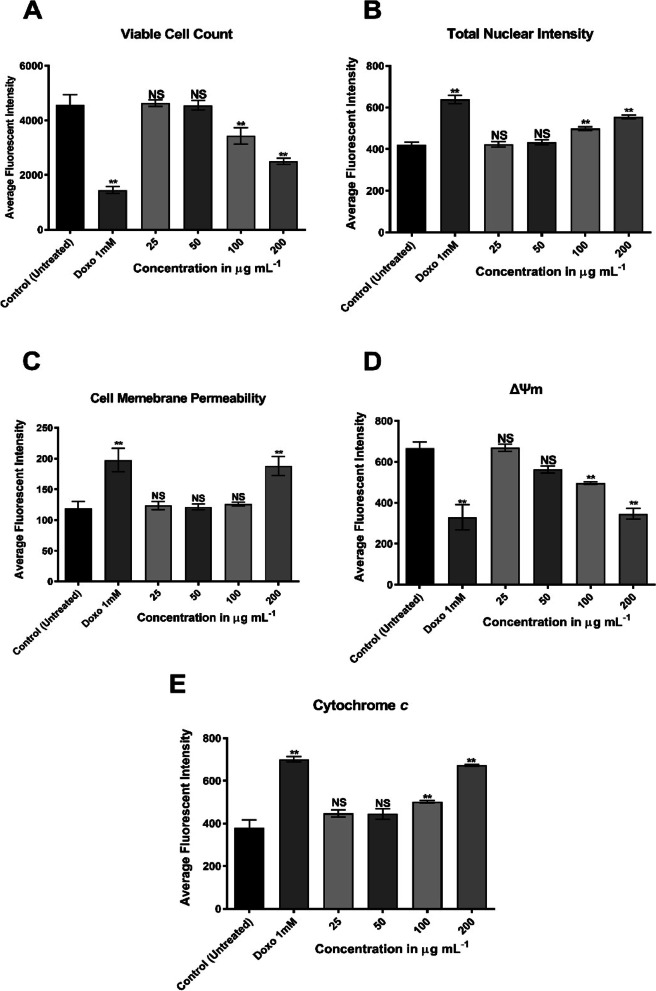
Mean (± SD) fluorescent intensity of MCF-7 cell count (**A**), cell nuclei (**B**), membrane permeability (**C**), mitochondrial membrane potential (**D**) and cytochrome C release (**E**), after treatment with AKBA (25, 50, 100 and 200 µg mL.^−^1) compared with untreated cells and doxorubicin treatment (1 mM) as positive control at 37 °C, 5% CO_2_ for 24 h. NS, non-significant, **p ≤ 0.01. SD, standard deviation (*n* = 3)

Figure 3A shows the viability of MCF-7 decreased by the increments of AKBA concentration in comparison with untreated cells. No notable changes in viability were detected in the 25 and 50 µg mL^−1^ treated cells; however, the viability of MCF-7 cells was significantly decreased at treatments 100 and 200 µg mL^−1^ (*p* = 0.0089 and 0.0004, respectively), with a reduced rate in cell count, reached up to 24.7 at 100 µg mL^−1^ and 45.14% at 200 µg mL^−1^. Exposure of MCF-7 cells to high AKBA concentrations (100 and 200 µg mL^−1^) caused changes in cell nuclear morphology by increased nuclear size and cell permeability. Cell staining with Hoechst 33,342 (blue) revealed a bright and condensed nuclear intensity with a 1.32-fold increase in response to 200 µg mL^−1^ treatment (Fig. 3B). On the other hand, the cell membrane intensity (green) was dramatically (*p* = 0.0037) affected in MCF-7 cells treated with 200 µg mL^−1^ AKBA in comparison with control untreated cells and with a 1.6-fold increase in intensity. No significant differences were recorded between doxorubicin treatment and AKBA treatment at a maximum applied concentration (Fig. 3C). A dose-dependent decline was observed in ΔΨm intensity by increasing AKBA concentration with a maximum reduction of 48.12% at 200 µg mL^−1^. Compared to the doxorubicin treatment (49.32% reduction), no significant differences were monitored with AKBA treatment at 200 µg mL^−1^. After being exposed to AKBA for 24 h, the fluorescence intensity in mitochondria was started to vanish by increasing the concentration, and the form of the fluorescence changed from filamentous spindle to spherical (Fig. 3D). The collapse of mitochondria significantly (*p* = 0.0034 and *p* ≤ 0.0001) triggered the translocation and release of cytochrome *c* from mitochondria into the cytosol in MCF-7 cells treated with AKBA at concentrations 100 and 200 µg mL^−1^, respectively. At 200 µg mL^−1^, AKBA activated the release of cytochrome C in a 1.8-fold increase compared with control (Fig. 3E).

### AKBA-induced apoptosis in MCF-7 cells

**Fig. 4:**
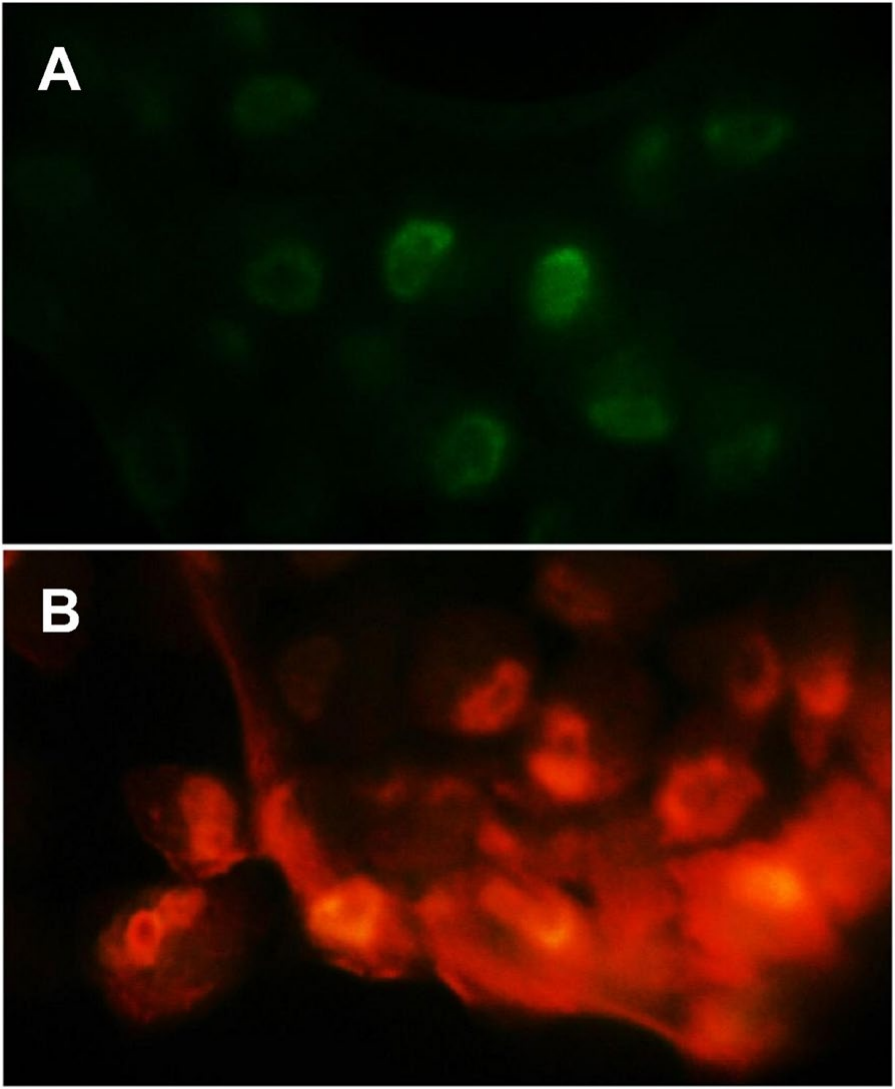
Fluorescence microscopic images of MCF-7 cells showing morphological changes after acridine orange-ethidium bromide dual staining. **A** Nontreated cells (control). **B** Cells treated by 100 µg mL^−1^ AKBA. Magnification 200 ×

The cytotoxic effect of AKBA at *IC*_50_ concentration (100 µg mL^−1^) against MCF-7 cells was examined using dual staining of acridine orange/ethidium bromide (AO/EtBr) and visualized in a fluorescent microscope. The untreated MCF-7 cells were observed with healthy, green and intact nuclei without distinguishable apoptotic cells (Fig. 4A). On the contrary, cells treated with AKBA displayed late stages of apoptosis indicated by intense and bright reddish colour (Fig. 4B).

### Generation of ROS

**Fig. 5:**
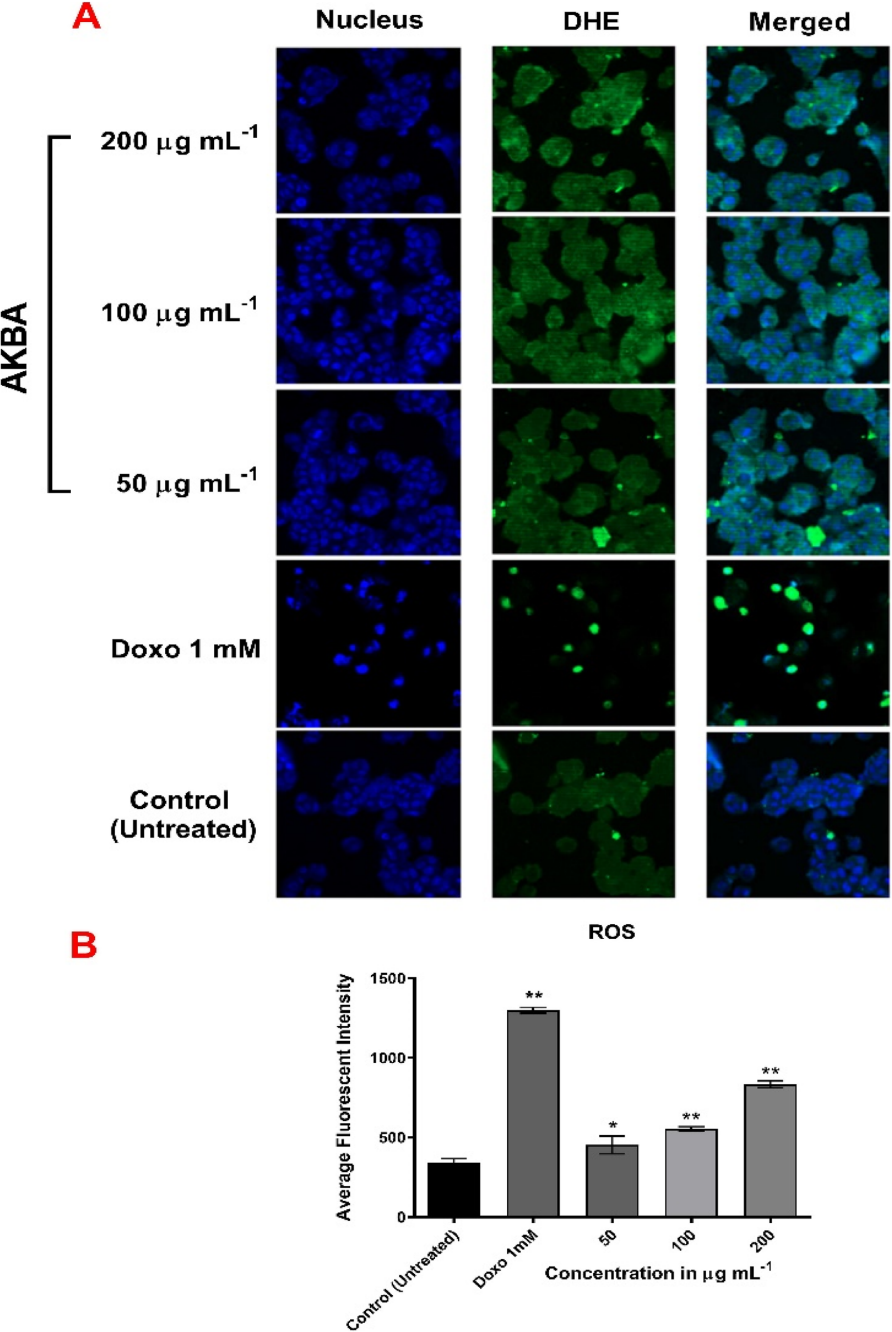
ROS generation. **A** Photographs of intracellular ROS production in MCF-7 cells treated with AKBA (50, 100 and 200 µg mL.^−1^) after 24 h of incubation at 37 °C, stained with DHE and visualized under HCS scan reader. **B** Representative histogram of mean (± SD) fluorescent intensity of DHE dye in MCF-7 cells treated with AKBA and doxorubicin at different concentrations. *p ≤ 0.05, **p ≤ 0.01. SD, standard deviation (*n* = 3)

Intracellular ROS generation by AKBA induction was monitored using HCS. As demonstrated in Fig. 5, exposure to increasing concentrations of AKBA significantly (*p* ≤ 0.01) increased the stain fluorescent intensity in a dose-dependent pattern which reflects strong production of ROS in MCF-7 cells. Compared with the untreated control, 1.32-, 1.61- and 2.44-fold ROS generation increases were achieved following 50, 100 and 200 µg mL^−1^ AKBA treatments, respectively.

### Estimation of caspase 8 and 9 activities

**Fig. 6:**
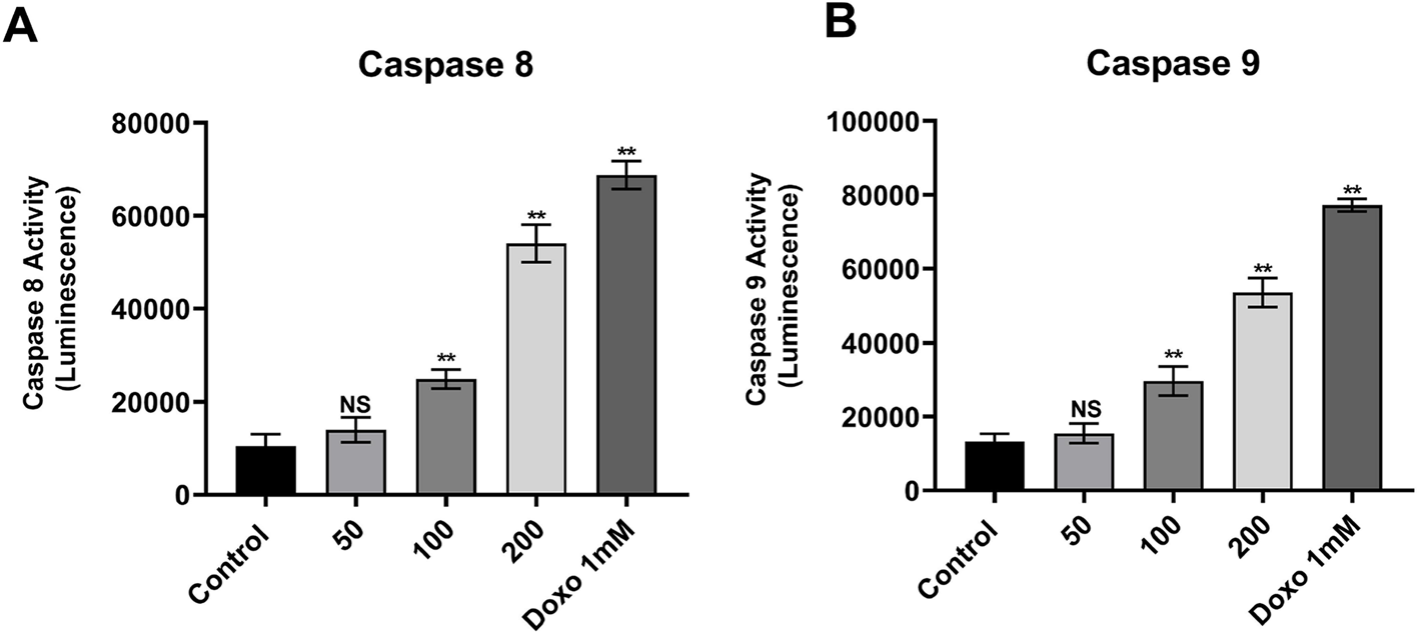
Mean (± SD) caspase 8 and 9 activities in MCF-7 cells. Activity measured in luminescence against various AKBA concentrations (50, 100 and 200 µg mL.^−1^) and positive control (doxorubicin 1 mM). Treatments were compared with control cells. NS, non-significant, *p ≤ 0.05, **p ≤ 0.01, SD standard deviation (*n* = 3)

The induction of caspases 8 and 9 was detected in MCF-7 cells treated with AKBA. Figure 6A shows a significant dose-dependent increase in the activity of caspase 8 detected in MCF-7 treated with AKBA at concentrations 100 (*p* = 0.0005) and 200 (*p* ≤ 0.0001) µg mL^−1^ for 24 h with induction activity up to 2.4- and 5.2-fold, respectively, compared with nontreated cells. In opposition, caspase 9 activity was also determined by examining MCF-7 cells treated with increasing concentrations of AKBA for 24 h. Results in Fig. 6B illustrate that the activity of caspase 9 significantly increased at AKBA concentrations 100 (*p* = 0.0002) and 200 (*p* ≤ 0.0001) µg mL^−1^ with 2.23- and 4.02-fold increase, respectively, compared with control cells.

### AKB-induced MCF-7 cell cycle arrest

**Fig. 7:**
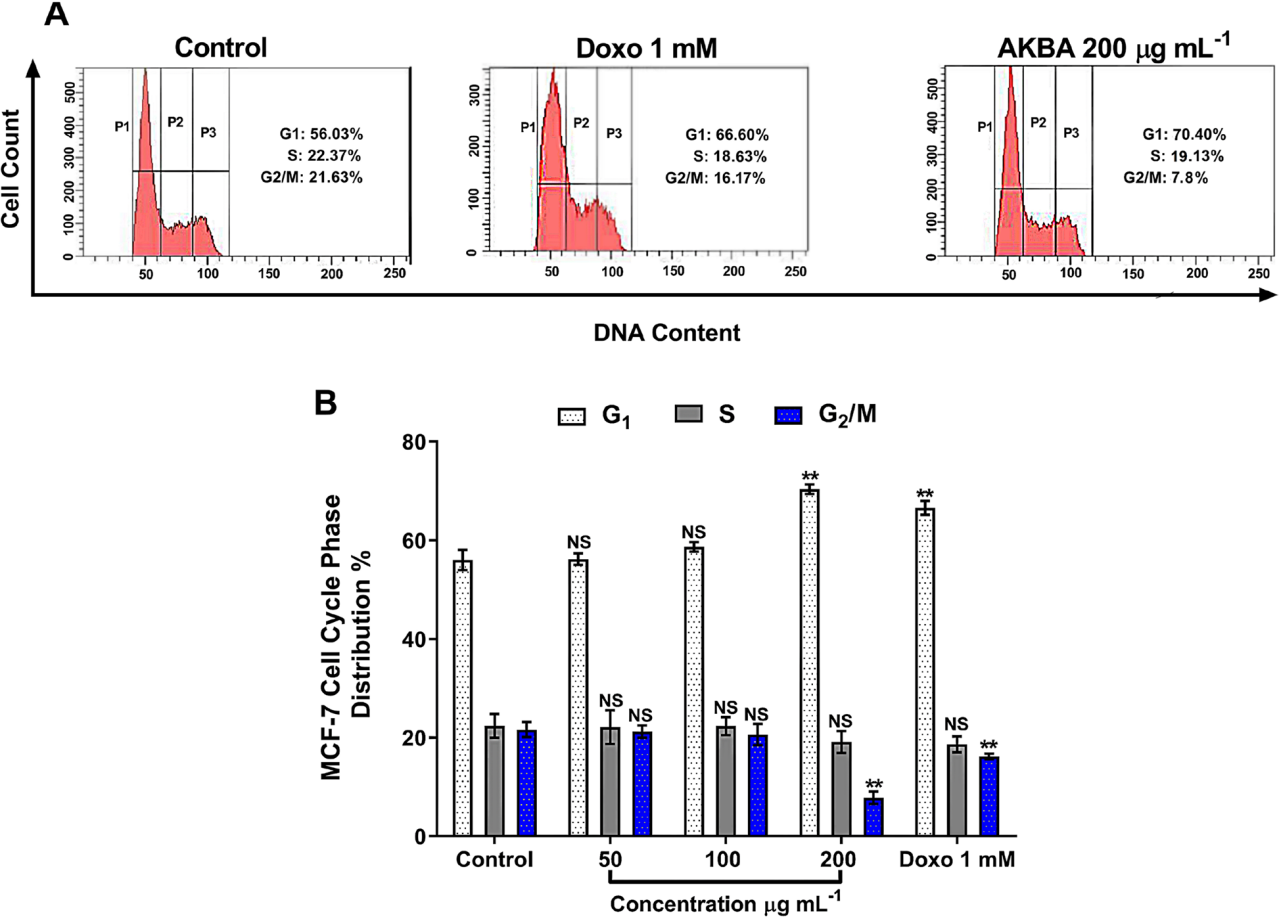
**A** Flow cytometric analysis and cell cycle distribution (G_1_, S and G_2_/M) of MCF-7 cells treated with AKBA (200 µg mL^−1^) and doxorubicin (1 mM) and were compared with untreated cells (negative control) for 24 h at 37 °C (*n* = 3). DNA content was evaluated with propidium iodide (PI) staining and fluorescently analysed. **B** Statistical analysis of cell cycle distribution mean percentage (± SD); differences for significance were made among the whole groups in each phase (G_1_, S and G_2_/M), **p < 0.01, NS, non-significant; SD, standard deviation

The cell cycle and phase distribution analysis of MCF-7 cells were conducted to assess the effect of AKBA concentrations (50, 100 and 200 µg mL^−1^) on the cell cycle compared with doxorubicin (1 mM) and untreated cells. As shown in Fig. 7, flow cytometric analysis exhibited that AKBA rarely caused changes in the percentage of all cell cycle phases compared with untreated cells at concentrations 50 and 100 µg mL^−1^. Interestingly, AKBA application at 200 µg mL^−1^ significantly (*p* ≤ 0.0001) increased the percentage of MCF-7 cell count (70.4%) at the G_1_ phase compared with untreated cells. By comparing with doxorubicin treatment, the accumulation of AKBA-treated cells (200 µg mL^−1^) at G_1_ showed no differences. The increase in cell count at G_1_ phase was accompanied by a sharp decrease (*p* ≤ 0.0001) of cell count at G_2_/M phase down to 7.8% as compared with the control.

## Discussion

*Boswellia serrata* is a medicinal plant known for its potential health characteristics in different parts of the world mostly in Asia. Due to its pharmacological activities such as anti-inflammatory, antiasthma and antidiarrheal, much interest was recently focused on *B. serrata* natural metabolites as novel and effective anticancer agents [7, 30]. AKBA, as a pentacyclic triterpenic acid, is considered as the most important natural constituent of *B. serrata* with promising therapeutic activity as a tumour antiproliferative agent [27].

According to MTT results, AKBA strongly inhibited MCF-7 cell proliferation. Many pathways for inhibiting tumour cells by AKBA were proposed. It was reported that using AKBA can activate the expression of *p52* and thus regulate the expression of cell cycle regulator *p21* gene and proapoptotic *Bax* gene which resulted in arresting HT-29 cells at the G_1_ phase, releasing cytochrome *c* and apoptosis [52]. Recently, AKBA acted by constraining topoisomerase I and II leading to DNA fragmentation and cell growth and proliferation inhibition [40]. Apoptosis induction via caspase activation-dependent pathway in colon, leukaemia, hepatoma and various other types of cancer via increased *Bax* expression, *NF-κB* lower expression and induction of poly(ADP)-ribose polymerase (PARP) cleavage was also determined [46]. Interestingly, β-BAs also interfere with protein synthesis by reacting with the ribosomal proteins and thus modulate cancer development [14]. Moreover, previous studies have shown the effectiveness of AKBA in the prevention and treatment of bladder, breast, colorectal, prostate, pancreatic and lung cancers [1, 24, 33, 49].

In the clonogenicity experiment, it was noted that the effect of AKBA against MCF-7 after 7 days was relatively dose dependent, confirming that AKBA can maximumly suppress cell proliferation and metastatic protentional at stably low doses suggesting that the continuous exposure to the AKBA at *IC*_50_ dose resulted in the significant killing of cancer cells. AKBA was reported to inhibit tumour cell proliferation and clonogenic formation via overexpression of cell cycle regulators that induce cell autophagy [39]. Relating to cell cycle arrest, AKBA was capable to undergo tumour cell apoptosis by promoting the expression of the *p21* gene, a protein associated with tumour cell arrest at G_1_ [17, 35].

Morphologically, changes in MCF-7 cells were monitored and quantitatively determined upon AKBA exposure using HCS. The count of MCF-7 cells was dropped by increasing the concentration of AKBA which could potentially be linked to the induction of programmed cell death. Cells with increased intensity of Hoechst blue stain contributed to the last stages of apoptosis which are characterized by cell shrinkage, nuclear compression, DNA fragmentation and the formation of apoptotic bodies [23]. Plasma membrane integrity loss is highly related to toxic or apoptotic effects [6]. High membrane permeability supports that AKBA at higher concentrations noticeably lead to cell membrane damage and consequently induced apoptosis since membrane permeability dye can only penetrate and stain cells with damaged membrane. Such observations are typically associated with various mechanisms of cell death including reshuffling events in plasma membrane leading to lose integrity [53].

Triggering of programmed cell death by mitochondrial membrane permeabilization, which describes as a mitochondrial (intrinsic) pathway, ultimately results in a reduction of ΔΨm, activation of a cascade of caspases, the release of cytochrome *c* and protein substrate cleavage and finally apoptosis [25, 26]. ΔΨm is an important indicator of functional mitochondria which is associated with membrane permeability of mitochondria. AKBA treatment at different concentrations significantly reduced ΔΨm in MCF-7 cells in a dose-dependent pattern. It was suggested that AKBA can modulate and highly disrupt the permeability transition pores of mitochondrial membrane and thus allowing the transition of molecules and ions, interrupting the respiratory chain, releasing cytochrome *c* and triggering apoptosis [32]. As previously reported, AKBA ameliorate its action against cancer cells by direct cleavage of (ADP-ribose) polymerases (PARP) which ultimately caused cancer cells to experience a cell cycle arrest, DNA fragmentation and a loss of ΔΨm [41]. Finally, cytochrome *c* release from the mitochondria of MCF-7 cells into the cytosol was observed due to AKBA treatments, especially at concentrations 100 and 200 µg mL^−1^. Upon release from the mitochondria into the cytoplasm, Cytochrome *c* has a notable role in programmed cell death, resulting in the activation of the caspase cascade and thereby commit the cell to death pathway. It was proposed that the release of cytochrome *c* is a result of the swelling and fracturing of the mitochondrial matrix which is then initiated by cell death stimulation [8].

AO/EtBr staining assay can identify apoptosis-related morphological changes in plasma membranes and nuclei during programmed cell death [34]. The results clearly showed that AO can normally penetrate intact MCF-7 cell membranes without AKBA treatment and emit a fluorescent green colour when bound with DNA. On the other hand, EtBr can enter only MCF-7-treated cells with the damaged cell membrane of apoptotic or dead cells and emitted orange-red fluorescent colour due to binding with fragmented and concentrated DNA, indicating late stages of cellular apoptosis [45].

Taken together, our results show that the excessive rate of ROS formation in MCF-7 cells was inversely correlated with dysfunctions in ΔΨm [31]. Destruction of mitochondrial integrity and induction of oxidative stress in MCF-7 cells imply a key toxicity mechanism. The outcomes of ROS induction by AKBA exposure lead to an explosion of cytochrome *c* from mitochondria into the cytosol and thus activating a cascade of apoptotic signalling pathways [10].

The molecular mechanism of AKBA in inducing apoptosis through the activation of effector caspase 8 and 9 was investigated. The treatment of MCF-7 cells with AKBA significantly stimulated the cleavage of caspases 8 and 9 precursors in a dose-dependent pattern, resulting in increased activation of both enzymes and thus induced intrinsic and extrinsic pathways of cell apoptosis. The increased level of caspase 8 highly suggested that treatment with AKBA activates caspase 8. Activation of caspase 8 is directly associated with a series of cellular events that leads to apoptosis starting with Bid protein truncation by caspase 8 into proapoptotic active protein (tBid) that links between the caspase 8 activation via receptor stimulation and mitochondrial cell death events [29]. After truncation of Bid into tBid, tBid instantly accumulates at the outer membrane of mitochondria, mediating an intrinsic series of apoptotic pathways including activation of *BAX*, the opening of mitochondrial anion channels and releasing of cytochrome *c* loss of mitochondrial membrane potential [13]. Depending on the results obtained, the increased level of caspase 8 and the decrease in MCF-7 mitochondrial membrane permeability with a significant release of cytochrome *c* were considered the hallmarks of AKBA treatments, which hardly suggested that AKBA can trigger the activation of caspase 8 which result in direct reduction of mitochondrial membrane potential. In addition, the release of cytochrome *c* into the cytosol and subsequent complexing and oligomerization of apoptotic protease activating factor-1 (Apaf-1) is related to the initiation of the intrinsic cell death pathway by the activation of caspase 9 which could lead to the activation of the caspase including caspase 7 [21]. Since caspase 9 was activated by AKBA, it is most likely that AKBA treatment in MCF-7 cells eventually mediated the mitochondrial cell death pathway. Confirmed by the observation of mitochondrial membrane disruption, cytochrome *c* release into the cytoplasm and subsequent elevation in caspase 9 level. Moreover, AKBA is considered a natural inhibitor of 5-lipoxygenase, a pro-inflammatory enzyme that catalyzes the biosynthesis of leukotrienes that is accountable for many diseases [12]. Recent findings revealed that the elevated expression of 5-lipoxygenase was significantly correlated with the high expression of more than 18 tumour-promoting genes in breast cancer and other types of cancer [19]. Inhibition of the 5-lipoxygenase pathway using different 5-lipoxygenase inhibitors including AKBA was recently demonstrated as a strategy for reducing tumorigenicity by promoting cell apoptosis [19, 38].

In addition to all mentioned above, cell cycle analysis was investigated, and it was found that AKBA at a concentration 200 µg mL^−1^ increased the cell count percentage at G_1_ phase, indicating that AKBA arrested MCF-7 cells at G_1_ phase and blocked the transition of cells from G_1_ to S phase with an apparent reduction in cell count percentage at G_2_/M phase. Of the cell cycle progression is a highly regulated process involving a combination of different proteins including cyclin-dependent kinases (CDKs) and cyclin-dependent inhibitors (CKI). Cyclin A and cyclin E along with CDK2 regulate the G_0_/G_1_ cell cycle phase and thus control DNA replication and mitosis in this phase. Arresting cells at the G_1_ phase and triggering apoptosis by AKBA treatment might be attributed to the suppression of cyclin A and/or cyclin E and upregulation of CKI that inhibit the combination between cyclin A and E with CDK2, therefore preventing the phase transition from G_1_ to S phase [35]. The activity of AKBA in arresting the tumour cell cycle at a particular phase was previously reported utilizing different tumour types. For instance, AKBA induced human lung cancer A549 arrest at G_0_/G_1_ [35, 36], while AKBA caused accumulation of HCT-116 cells, a colon cancer cell line, in the G_2_/M phase, promoting cell arrest at G_2_/M [48].

## Conclusion

Based on our findings, we can conclude the following: AKBA demonstrated a cytotoxic capability against MCF-7 cells in a dose-dependent pattern, compared with the normal MCF-10A cells with the ability to inhibit clone formation. AKBA significantly decreased cell viability and increased membrane permeability, reduction in ΔΨm and activation of mitochondrial-dependent proapoptotic cytochrome *c* release. Also, MCF-7 cells exhibited late stages of cellular apoptosis with morphological changes in the nucleus and cell membrane. The reduction in cell viability was accompanied by a significant production of intracellular ROS levels. These events triggered the increased activation of caspase 8 and 9 and finally arrested the MCF-7 cell cycle at the G_1_ phase.

## Data Availability

All the data supporting the findings of this research are available in the article.
